# Effect of *Diashis*, a polyherbal formulation, in streptozotocin-induced diabetic male albino rats

**DOI:** 10.4103/0974-7788.59939

**Published:** 2010

**Authors:** Tushar K. Bera, Debasis De, Kausik Chatterjee, Kazi M. Ali, Debidas Ghosh

**Affiliations:** 1 *Department of Bio-Medical Laboratory Science and Management, (U.G.C. Innovative Programme Funded Department), Vidyasagar University, Midnapore - 721 102, India*; 2 *Ayurvedic Division, Southern Health Improvement Samity (SHIS), Bhangar, South 24 - Paraganas, West Bengal, India*

**Keywords:** Antihyperglycemic, antioxidative, *Diashis*

## Abstract

This study focuses on the effect of ‘*Diashis*’, a polyherbal formulation composed of eight medicinal plants for the management of streptozotocin (STZ)-induced diabetes in rats. As oxidative stress is one of the consequences of diabetes, the activities of hepatic antioxidant enzymes and metabolic enzymes were evaluated. Treatment with ‘*Diashis*’ in STZ-induced diabetic rats resulted in a significant (*P* < 0.01) recovery in the activities of hepatic hexokinase, glucose-6-phosphate dehydrogenase, and glucose-6-phosphatase along with correction in the levels of fasting blood glucose, glycated hemoglobin, and liver and skeletal muscle glycogen. The oxidative stress status in the liver was corrected by ‘*Diashis*’ which was highlighted by the recovery in the activities of catalase, peroxidase, and glutathione-*S*-transferase along with the correction in the quantity of thiobarbituric acid-reactive substances and conjugated diene. ‘*Diashis*’ was not found to have any metabolic toxicity. The antidiabetic effects of ‘*Diashis*’ were compared with those of the antidiabetic drug, ‘Glibenclamide’.

## INTRODUCTION

Polyherbal drugs are considered to be more effective for the management of diabetes with Ayruvedic medicines.[1,2] ‘*Diashis*’, a polyherbal proprietary formulation, consists of eight plants, *i.e., Syzygium cumuni, Gymnema sylvestre, Holarrhena antidysenterica, Tinospora cordifolia, Pongamia pinnata, Asphultum, Psoralea corylifolia*, and *Momordica charantia*. Each of these plants has been reported to have antidiabetic and antioxidative activities [Table 1].[3–11] Although ‘*Diashis*’ is used for the management of diabetes, not much is known about the scientific basis of this use or its general toxicity. WHO gives special emphasis to herbal antidiabetic drug development with proper screening.[12]

**Table 1:** Composition of ingredient(s) present in polyherbal formulation, ‘*Diashis*’

Botanical name	Common name	Family	Part used	Ingredients of ‘*Diashis*’ (stock sample) *(mg)
*Syzygium cumuni*	Jaam	Myrtaceae	Seed	50
*Gymnema sylvestre*	Meshasringi	Asclepiadaceae	Leaves	75
*Holarrhena antidysenterica*	Indrayab	Apocynaceae	Seed	50
*Tinospora cordifolia*	Guduchi	Menispermaceae	Root	75
*Pongamia pinnata*	Natakaranja	Fabaceae	Seed	100
*Asphultum*	Silajit	Pedaliaceae	Gum	50
*Psoralea corylifolia*	Somraji	Fabaceae	Seed	50
*Momordica charantia*	Karala	Cucurbitaceae	Seed	50

* This stock sample was used at the dose mentioned in the experiment

## MATERIALS AND METHODS

### Preparation of the polyherbal formulation, ‘*Diashis*’

The eight medicinal plants mentioned above which were used for the preparation of the polyherbal formulation, ‘*Diashis*’, were provided to us by the ‘Ayurvedic Division of Southern Health Improvement Samity (SHIS)’, 24-Parganas (S), West Bengal, India. These plants were confirmed by the Botany Department in Vidyasagar University. Herbal specimens were preserved in the Departmental Herbarium Museum as SC-Bio-Med-1/9, GS-Bio-Med-2/9, HA-Bio-Med-3/9, TC-Bio-Med-4/9, PP-Bio-Med-5/9, As-Bio-Med-6/9, PC-Bio-Med-7/9, and MC-Bio-Med-8/9.

The desired parts of eight medicinal plants [Table 1] were dried in an incubator for 24 h at 37°C, crushed separately in an electrical grinder, and pulverized. The powdered forms of the relevant parts of these medicinal plants were then mixed in fixed ratios as per Table 1 and referred to as ‘*Diashis*’. The polyherbal formulation of ‘*Diashis*’ was prepared on the basis of an Ayurvedic antidiabetic formulation described in some reports.[13]

### Diashis administration

‘*Diashis*’ powder was dissolved in distilled water and the diabetic rats were orally treated by forceful gavage with a dose of 5 mg/0.5 mL distilled water/100 g body weight/rat/day in the fasting state for 21 days. This dose was selected from our pilot study using doses starting from 2 mg up to 20 mg/100 g body weight wherein the above dose (5 mg/100 g body weight) was noted as the threshold dose. In traditional medicine, the dose of ‘*Diashis*’ given to humans has been reported to be 20–30 mg/kg body weight (2–3 mg/100 g body weight/day).

### Selection of animals and animal care

The study was conducted on mature Wistar strain male albino rats, three months of age, weighing about 150 ± 10 g. Animals were acclimated for a period of fifteen days in our laboratory conditions prior to the experiments. Rats were housed in tarsons cages (six rats per cage), at an ambient temperature of 25 ± 2°C with 12 h light: 12 h dark cycle. The rats had free access to standard food and water. Permission was obtained from the Animal Ethical Committee (AEC) for this experiment. The Principles of Laboratory Animal Care were followed throughout the duration of the experiment and instructions given by our Institutional Ethical Committee were followed regarding treatment during the experiments (NIH, 1985). Normoglycemic animals were selected for this experiment as having fasting blood glucose levels of 75 ± 5 mg/dL.[5]

### Chemicals

Streptozotocin (STZ) was purchased from Sigma, USA. Other chemicals were purchased from Sigma-Aldrich Diagnostic Ltd. USA or from SRL, India. Biochemical kits were purchased from Span Diagnostic Ltd., Surat, India.

### Induction of diabetes mellitus

Diabetes was induced with streptozotocin as indicated by our standard method mentioned earlier.[14] Briefly, 24 h fasting rats were given 4 mg streptozotocin (STZ)/0.1 mL of citrate buffer (pH 4.5)/100 g body weight/rat as a single intramuscular injection to produce type-I diabetes after 24 h of the injection.[15] For seven successive days, their diabetic state was monitored for its stability. Eighteen rats with stable diabetes having fasting blood glucose levels ≥250 mg/dL were selected as moderately diabetic rats for this experiment.

### Experimental Design

Rats were divided into the following four equally sized groups for 21 days of the study. ‘*Diashis*’ was administered for 21 days after confirmation of a stable diabetic state in STZ-injected rats. Twenty-one days were chosen as duration of treatment because this was the threshold duration of treatment in our pilot experiments.

Group I: Untreated Control received 0.5 mL of distilled water/100 g body weight/rat/day for 21 days by gavage forcefully. Six normoglycemic animals were included in this group.

Group II: Diabetic  Control rats were made diabetic as mentioned before. Six diabetic rats were included here and 0.5 mL distilled water was provided forcefully/100 g body weight/rat/day for 21 days.

Group III: Diabetic + *Diashis* diabetic rats were forcefully fed by gavage with ‘*Diashis*’ at a dose of 5 mg/0.5 mL of distilled water/100 g body weight/rat/day early in the morning and in fasting condition for 21 days.

Group IV: Diabetic + Glibenclamide diabetic rats were given 2 mg Glibenclamide/0.5 mL distilled water/100 g body weight/ rat/day forcefully by gavage for 21 days in the fasting state.

Starting from the first day of treatment with the polyherbal formulation in these diabetic rats, fasting blood glucose (FBG) levels of all the groups were measured by using a single-touch glucometer every seven days. On the 22^nd^ day of the experiment (29^th^ day from the day of STZ injection), all the animals were sacrificed after recording the final body weight. Blood was collected from the dorsal aorta by using a syringe. Serum was separated from a part of the collected blood by centrifugation at 3000 × g for 5 min for the evaluation of SGPT and SGOT activities. The remaining blood was used for the quantification of glycated hemoglobin (HbA_1c_). The liver and skeletal muscles were dissected out and stored at – 20°C. The activities of key carbohydrate metabolic enzymes, *i.e*., hexokinase, glucose-6-phosphate dehydrogenase, and glucose-6-phosphatase were measured biochemically. The activities of antioxidative enzymes (catalase (CAT), peroxidase (Px), glutathione-*S*-transferase (GST)) were measured besides the quantification of conjugated diene (CD) and thiobarbituric acid-reactive substances (TBARS) in the liver for the assessment of oxidative injury on metabolic organs. Glycogen content was also determined in the liver and the skeletal muscle.

### Measurement of fasting blood glucose level

Fasting blood glucose (FBG) levels were measured at the time of grouping of the animals. After every seven days of treatment, FBG levels were recorded from all the animals of all the groups. Blood was collected from the tip of the tail vein or by orbital puncture and FBG levels were measured by using a single-touch glucometer.

### Glycated hemoglobin (HbA_1c_) level

Glycated hemoglobin (HbA_1c_) was measured according to a standard protocol.[16] For this purpose, 4 mL of blood was collected in an EDTA-containing bulb and plasma was separated. The packed cell pellet was washed six times with normal saline (0.9% NaCl). The hemolysate was prepared by adding 1/4^th^ part of distilled water and 1/4^th^ part of carbon tetrachloride to the packed cell pellet and centrifuging at 3000 r.p.m for 20 min. Hemoglobin concentration of the hemolysate was measured by using the cyanmethemoglobin method and it was adjusted to 10 mg/dL by using normal saline. Two milliters of 10 mg/dL hemoglobin-containing hemolysate were taken to which was added 1.0 mL of 0.3N oxalic acid and mixed. The mixture was kept in a boiling water bath for one hour, then cooled to room temperature, and 1 mL of 40% TCA was added to this mixture. The total content was mixed and centrifuged at 3000 r.p.m. Two milliters of the supernatant were collected and 0.5 mL of 0.7% thiobarbituric acid was added and kept at 37°C for 40 min. Absorbances were noted against the blank consisting of 2 mL distilled water and 0.5 mL thiobarbituric acid at 443 nm. The glycated hemoglobin level was expressed as GHb%.

### Biochemical assay of hexokinase

Hexokinase activity in liver tissue was determined spectrophotometrically as follows:[17] The assay mixture contained 3.7 mM glucose, 7.5 mM MgCl_2_, 11 mM thioglycerol, and 45 mM HEPES buffer. Tissue was homogenized in ice-cold 0.1M phosphate-buffered saline (pH 7.4) to a concentration of 50 mg/mL. Into a spectrophotometer cuvette were added 0.9 mL of this assay mixture and 0.03 mL of 0.22M ATP and mixed well. After that, 0.1 mL of the tissue supernatant was added into the cuvette and absorbance noted at 340 nm. One unit of hexokinase was expressed as μg/mg of tissue.

### Biochemical assay of glucose-6-phosphate dehy drogenase

Liver glucose-6-phosphate dehydrogenase activity was measured spectrophotometrically as per our modified protocol.[18] One unit of enzyme activity is defined as the quantity that catalyzes the reduction of 1 µM of NADP per minute. Activity of this enzyme was recorded by using glucose-6-phosphate as a substrate and the absorbance was measured at 340 nm.[19]

### Biochemical assay of glucose-6-phosphatase activity

Liver glucose-6-phosphatase activity was measured according to the standard protocol.[20] Tissue was homogenized in ice-cold 0.1M phosphate-buffered saline (pH 7.4) to a concentration of 50 mg/mL. In a calibrated centrifuge tube, 0.1 mL of 0.1M glucose-6-phosphate solution and 0.3 mL of 0.5M maleic acid buffer (pH 6.5) were taken and incubated in a 37°C water bath for 15 min. The reaction was stopped by the addition of 1 mL of 10% trichloroacetic acid (TCA), followed by chilling in ice and centrifuging at 3000 × g for 10 min. The optical density was noted at 340 nm and the enzyme activity was expressed as mg of inorganic phosphate liberated/g of tissue.

### Biochemical assay of glycogen content

Glycogen content in liver and skeletal muscle was measured biochemically.[21] Tissues were homogenized in 80% ethanol and the extracts collected by centrifugation using anthrone reagent. The quantity of glycogen was measured in relation to the standards and expressed in μg of glucose/mg of tissue.

### Biochemical assay of catalase activity

The activity of catalase in the hepatic tissue was measured biochemically.[22] For the evaluation of catalase activity, liver tissue was homogenized in 0.05 M Tris-HCl buffer solution (pH 7.0) to a concentration of 50 mg/mL. These homogenized samples were centrifuged at 10000 × g at 4°C for 10 min. In a spectrophotometer cuvette, 0.5 mL of 0.00035M H_2_O_2_ and 2.5 mL of distilled water were added and mixed. Readings of absorbance were noted at 240 nm before the addition of supernatant. Supernatant from the sample was added at a volume of 40 μL to the cuvette and the subsequent six readings were noted at 30 sec interval.

### Biochemical assay of peroxidase activity

The peroxidase activity was measured in hepatic tissue according to the standard method.[23] The samples were homogenized in ice-cold 0.1M phosphate-buffered saline (pH 7.0) to a concentration of 50 mg/mL. Next, 20 mM guiacol was mixed with 0.1 mL supernatant collected from the homogenate. In the presence of 0.3 mL of 12.3 mM H_2_O_2_, the time taken for an increase in the absorbance by 0.1 units was recorded at 436 nm.

### Estimation of glutathione-S-transferase activity

Activity of GST in the liver tissue was measured spectro photometrically using 1-chloro-2,4-dinitrobenzene (CDNB) as a substrate.[24] The assay mixture was 3 mL of a mixture containing 0.1 mL of 1 mM CDNB in ethanol, 0.1 mM of 1 mL GSH, 2.7 mL of 100 mM potassium phosphate buffer (pH 6.5), and 0.1 mL of supernatant of the tissue homogenate. The formation of the product of CDNB-*S*-2,4- dinitrophenylglutathione was monitored by measuring the net increase in absorbance at 340 nm against the blank. The enzyme activity was calculated using the extinction coefficient, 6.9 M/cm and expressed in unit/mg of tissue.

### Estimation of lipid peroxidation from concentration of thiobarbituric acid-reactive substances and conjugated diene

Liver tissue was homogenized to a concentration of 50 mg/mL in 0.1 M of ice-cold phosphate buffer (pH 7.4) and the homogenate was centrifuged at 10000 × *g* at 4°C for 5 min. Each supernatant was used for the estimation of TBARS and CD. For the measurement of TBARS, 0.5 mL of the supernatant was mixed with 0.5 mL of normal saline (0.9% NaCl) and 2 mL of TBA- TCA mixture (0.392 g thiobarbituric acid in 75 mL of 0.25N HCl with 15 g trichloroacetic acid). The volume of the mixture was made up to 100 mL with 95% ethanol and boiled at 100°C for 10 min. This mixture was then cooled to room temperature and centrifuged at 4000 × g for 10 min. The whole supernatant was taken into a spectrophotometer cuvette and read at 535 nm.[25]

Quantification of CD was performed by a standard method.[26] The lipids were extracted with a chloroform-methanol (2:1) mixture, followed by centrifugation at 1000 × g for 5 min. The chloroform layer was evaporated to dryness under a stream of nitrogen. The lipid residue was dissolved in 1.5 mL of cyclohexane and the absorbance noted at 233 nm to measure the amount of hydroperoxide formed.

### Biochemical assay of serum glutamate oxaloacetate transaminase and serum glutamate pyruvate transaminase activities

The activities of glutamate oxaloacetate transaminase (GOT) and glutamate pyruvate transaminase (GPT) in serum were measured by using specific kits supplied by Span Diagnostic Ltd. Surat; India. The activities of these enzymes were expressed as IU/L of serum.[27]

### Statistical analysis

Analysis of Variance (ANOVA) followed by multiple comparison Student's two-tail ‘*t*’-test was used for statistical analysis of collected data.[28] Differences were considered to be significant at the levels of *P* < 0.01 and *P* < 0.001. All the values indicated in the tables are Mean ± SD values.

## RESULTS

### Body weight

Body weights were found to be significantly decreased (*P* < 0.001) in STZ-induced diabetic rats with respect to the control group. Treatment with ‘*Diashis*’, a polyherbal formulation, resulted in a significant recovery of body weight loss towards the control level (*P* < 0.01). No difference was noted in body weights between ‘Glibenclamide’-treated group and ‘*Diashis*’-treated group [Table 2].

**Table 2:** Effect of ‘*Diashis*’, a polyherbal formulation, on body weight, hemoglobin and glycated hemoglobin levels in streptozotocin-induced diabetic male albino rats

Groups	Body weight (g)		Hemoglobin (g/dL)	Glycated hemoglobin (GHb %)
	
	Initial	Final		
Untreated control	154.76 ± 8.51	158.03 ± 8.54	14.21 ± 0.91	1.75 ± 0.13
Diabetic control	155.34 ± 9.70	138.52 ± 6.41*	5.56 ± 0.51*	3.76 ± 0.47*
Diabetic + *Diashis*	153.87 ± 10.40	151.01 ± 7.90**	11.04 ± 0.87**	2.06 ± 0.20**
Diabetic + Glibenclamide	151.26 ± 5.36	152.22 ± 5.14**	11.83 ± 0.89**	1.98 ± 0.17**

All the values are expressed as Mean ± SD, *n* = 6. ANOVA followed by multiple comparisons two-tail ’*t*’-test where

* indicates *P* < 0.001 and

** indicates *P* < 0.01 compared with untreated control group

### Hemoglobin (Hb) and glycated hemoglobin (HbA_1c_) levels

Glycated hemoglobin (HbA_1c_) levels were found to be significantly increased (*P* < 0.001) and hemoglobin (Hb) levels had decreased significantly (*P* < 0.001) in the STZ-induced diabetic group with respect to the control group. Significant recovery (*P* < 0.01) were noted in the Hb and HbA_1c_ levels towards the control levels after treatment with ‘*Diashis*’. No difference was noted in levels of Hb and HbA_1c_ in the ‘Glibenclamide’-treated group and the ‘*Diashis*’-treated group [Table 2].

### Fasting blood glucose level

STZ-induced diabetic animals showed a significant (*P* < 0.001) elevation in fasting blood glucose (FBG) levels in comparison to the control group. Treatment with ‘*Diashis*’ for 21 days resulted in a significant (*P* < 0.01) recovery of FBG levels in the diabetic animals towards control levels. There was no difference between ‘Glibenclamide’-treated and ‘*Diashis*’-treated groups [Table 3].

**Table 3:** Corrective effect of ‘*Diashis*’, a polyherbal formulation, on fasting blood glucose level in streptozotocin-induced diabetic male albino rats

Groups	Fasting blood glucose level (mg/dL)		
	
	0 day	1^st^ day	28^th^ day
Untreated control	74.2 ± 9.39	73.21 ± 10.06	72.1 ± 9.84
Diabetic control	72.21 ± 10.96	299.7 ± 9.62*	382.52 ± 12.52*
Diabetic + *Diashis*	73.61 ± 11.41	298.52 ± 10.06*	98.02 ± 8.72**
Diabetic + Glibenclamide	75.61 ± 11.51	294.73 ± 10.28*	95.12 ± 10.50**

All the values are expressed as Mean ± SD, *n* = 6. ANOVA followed by multiple comparisons two-tail ’*t*’-test where

* indicates *P* < 0.001 and

** indicates *P* < 0.01 compared with untreated control group

### Carbohydrate metabolic enzymes

Significant (*P* < 0.001) decrease in the activities of hepatic hexokinase and glucose-6-phosphate dehydrogenase, and elevation in the activity of glucose-6-phosphatase were noted in the STZ-induced diabetic group with respect to the control group [Table 4]. After treatment with ‘*Diashis*’ in the diabetic rat, significant (*P* < 0.01) recovery was noted in the activities of the said enzymes. No significant difference was noted in the activities of the concerned enzymes between ‘*Diashis*’-treated and ‘Glibanclamide’-treated groups [Table 4].

**Table 4:** Effect of ‘*Diashis*’ on the activities of key enzymes of carbohydrate metabolism in liver, and glycogen content in liver and skeletal muscle in streptozotocin-induced diabetic rats

Groups	Hexokinase (µg/mg of tissue)	Glucose-6-phosphate dehydrogenase (unit/mg of tissue)	Glucose-6-phosphatase (mg of IP/g of tissue)	Glycogen content (μ of glucose/mg of tissue)	
				
				Liver	Muscle
Untreated control	140.19 ± 6.73	14.66 ± 0.25	19.66 ± 2.62	46.7 ± 2.68	36.5 ± 2.01
Diabetic control	109.78 ± 5.63*	6.15 ± 1.45*	33.91 ± 1.45*	26.1 ± 2.91*	22.15 ± 2.68*
Diabetic + *Diashis*	131.46 ± 4.34**	11.04 ± 1.65**	24.83 ± 2.68**	38.3 ± 3.58**	30.17 ± 2.92**
Diabetic + Glibenclamide	129.07 ± 4.36**	10.97 ± 1.49**	23.96 ± 2.21**	40.22 ± 4.02**	32.04 ± 3.08**

All the values are expressed as Mean ± SD, *n* = 6. ANOVA followed by multiple comparisons two-tail ’*t*’-test where

* indicates *P* < 0.001 and

** indicates *P* < 0.01 compared with untreated control group

### Glycogen level in tissues

Levels of glycogen were seen to be significantly decreased in the liver and skeletal muscle (*P* < 0.001) of the diabetic group compared to the control group. Treatment with ‘Glibencalmide’ or ‘*Diashis*’ resulted in a significant (*P* < 0.01) recovery in the levels of glycogen in liver and skeletal muscle towards the control levels [Table 4].

### Activities of catalase, peroxidase and glutathione-S- transferase

Activities of hepatic CAT, Px and GST were found to be significantly decreased (*P* < 0.001) in the diabetic group with respect to the control group. Treatment with ‘*Diashis*’ resulted in a significant (*P* < 0.01) elevation in the activities of above mentioned enzymes towards their control levels. No difference was noted in the activities of these enzymes between the ‘Glibenclamide’-treated and ‘*Diashis*’- treated groups [Table 5].

**Table 5:** Remedial effect of ‘*Diashis*’ on the activities of hepatic antioxidant enzymes and levels of lipid peroxidation in streptozotocin-induced diabetic rats

Groups	Antioxidant enzymes activities			Lipid peroxidation levels	
		
	CAT (mM of H_2_O_2_ consumption/mg of tissue/min)	Px (unit/mg of tissue)	GST (unit/mg of tissue)	TBARS (nM/mg of tissue)	CD (nM/mg of tissue)
Untreated control	3.61 ± 0.18	3.73 ± 0.35	1.05 ± 0.11	28.63 ± 2.72	243.10 ± 6.08
Diabetic control	1.79 ± 0.11*	1.12 ± 0.25*	0.29 ± 0.06*	42.28 ± 4.24*	319.54 ± 11.94*
Diabetic + *Diashis*	2.89 ± 0.13**	2.82 ± 0.29**	0.83 ± 0.08**	33.01 ± 3.44**	272.43 ± 13.59**
Diabetic + Glibenclamide	2.87 ± 0.11**	2.94 ± 0.31**	0.88 ± 0.06**	36.46 ± 3.21**	246.98 ± 5.92**

All the values are expressed as Mean ± SD, *n =* 6. ANOVA followed by multiple comparisons two-tail ’*t*’-test where

* indicates *P* < 0.001 and

** indicates *P* < 0.01 compared with untreated control group

### Conjugated diene and thiobarbituric acid reactive substances levels

Liver levels of CD and TBARS were seen to be significantly increased (*P* < 0.001) in the diabetic group when compared with the control group. After the treatment with ‘*Diashis*,’ there was a significant recovery (*P* < 0.01) in the liver levels of TBARS and CD of the diabetic rats towards the control levels. Levels of TBARS and CD in the ‘Glibenclamide’-treated group were not different from those of the ‘*Diashis*’-treated group [Table 5].

### Activities of glutamate oxaloacetate transaminase and glutamate pyruvate transaminase in serum

Activities of GOT and GPT in serum were found to be significantly increased (*P* < 0.001) in the diabetic group compared to the control group. Treatment with ‘*Diashis*’ resulted in a significant (*P* < 0.01) recovery in the levels of these two parameters towards the control levels. No difference was noted in the activities of serum GOT and GPT between the ‘Glibenclamide’-treated and ‘*Diashis*’- treated groups [Table 6].

**Table 6:** Corrective effect of ‘*Diashis*’, on serum transaminase activities in streptozotocin-induced diabetic male albino rats

Groups	Serum transaminase activities (IU/L)		
	
	SGPT	SGOT
Untreated control	78.02 ± 3.04	38.33 ± 4.50
Diabetic control	111.89 ± 4.72*	60.32 ± 4.87*
Diabetic + *Diashis*	89.08 ± 4.18**	47.64 ± 4.74**
Diabetic + Glibenclamide	87.52 ± 4.69**	49.07 ± 4.87**

All the values are expressed as Mean ± SD, *n* = 6. ANOVA followed by multiple comparisons two-tail ’*t*’-test where

* indicates *P* < 0.001 and

** indicates *P* < 0.01 compared with untreated control group

## DISCUSSION

The present study was conducted to determine the antihyperglycemic as well as antioxidative effects of ‘*Diashis*’, a polyherbal formulation, in STZ-induced diabetic male albino rats. The effects were compared with those of the standard antidiabetic drug, ‘Glibenclamide’. *Diashis* is an Ayurvedic polyherbal formulation that is widely used for the treatment of diabetes in some areas of West Bengal, however, its mechanism of antidiabetic action is not known. Hence, we studied fasting blood glucose (FBG) levels along with glycogen content in liver and skeletal muscle, and the activities of some important carbohydrate metabolism enzymes. Moreover, we have also assessed the stress-induced oxidative status in the livers of the experimental groups because diabetes has a strong association with oxidative injury.[29]

This study selected the STZ-induced diabetic rat as an experimental model because it is commonly used model to study the effects of antidiabetogenic agents.[30,31] Streptozotocin-induced diabetes has been demonstrated in this study by the decrease in the activities of hexokinase and glucose-6-phosphate dehydrogenase as these enzymes are regulated by insulin.[32] These results are consistent with those already reported by our previous publication.[33] Diabetes induction has been strengthened further by the elevated activity of glucose-6-phosphatase as this is under the negative control of insulin.[34] Treatment with ‘*Diashis*’ or ‘Glibenclamide’ resulted in a significant (*P* < 0.001) recovery in the activities of the said enzymes which may be due to the recovery in plasma insulin levels. Another possibility for the decrease in the activities of hexokinase and glucose-6-phosphate dehydrogenase in diabetes may be due to diabetes induced-oxidative injury as free radicals are scavengers of structural and functional protein including enzyme in cells.[35] This has been reflected here by the significant decrease in the activities of key antioxidant enzymes like catalase (CAT), peroxidase (Px), and glutathione-*S*-transferase (GST) in the liver.[36] The decrease in the activities of antioxidant enzymes in diabetes may be due to low levels of insulin or due to high levels of advanced glycated end products.[37,38] This hypothesis has been strengthened by the elevation in the levels of end products of free radicals like TBARS and CD, which has also been supported by our previous publication and also by others.[39,40] Significant recovery in the activities of antioxidant enzymes due to ‘*Diashis*’ may be due to correction in blood glucose levels or by plasma insulin through ß-cells stimulating effect of phyto-ingredient(s) present in ‘*Diashis*’ as claimed by others by using others plant parts.[41] The correction in diabetes induced-oxidative injury by ‘*Diashis*’ was supported by the decrease in the levels of CD and TBARS, the end products of free radical generation.[42]

The antidiabetic effect of *Diashis* has been further supported here by the measurement of glycated hemoglobin (HbA_1c_) as proposed by others and by our previous reports.[43,44] Treatment with ‘*Diashis*’ or ‘Glibenclamide’ in diabetic rats resulted in a significant recovery in hemoglobin and HbA_1c_ levels, which may be due to recovery in plasma glucose towards the control levels.

Quantification of glycogen in the liver and skeletal muscle further confirms the efficacy of ‘*Diashis*’ for the recovery of diabetes, which, in turn, supports the insulinotropic effect of ‘*Diashis*’, as insulin is the main regulator of glycogen content in liver and skeletal muscle.[45]

This polyherbal formulation has no general toxic effect as body weights remain similar to those in the control and the ‘*Diashis*’-treated groups. Moreover, there was no change in the activities of serum GOT and GPT in the polyherbal formulation-treated group which also illustrates the nontoxic effect of ‘*Diashis*’.

In conclusion, the polyherbal formulation ‘Diashis’ appears to compare favourably with glubln alamide drug.
